# Management of vision loss associated with complications of cosmetic filler injections

**DOI:** 10.3389/fopht.2025.1568370

**Published:** 2025-04-11

**Authors:** Samantha Madala, Shaili Davuluru, Joy Li, Jeffrey Gluckstein, John Martin, Kasra Khatibi, Sandy Zhang-Nunes

**Affiliations:** ^1^ University of Southern California (USC) Roski Eye Institute, Keck School of Medicine of the University of Southern California, Los Angeles, CA, United States; ^2^ Independent Practitioner, Coral Gables, FL, United States

**Keywords:** cosmetic fillers, injectable fillers, filler complications, orbital ischemia, ocular ischemia

## Abstract

Injectable cosmetic fillers have dramatically risen in popularity in recent years. However, as the use of such fillers has become more common, there have been many reports of vision loss secondary to misplaced filler embolizing to the ophthalmic artery resulting in ocular ischemia. Currently, there are no randomized control trials or widely validated clinical guidelines that dictate how injectors should manage ischemic complications of filler embolism. This review aims to explain the possible mechanisms by which a cosmetic filler embolus can occlude the ophthalmic artery, describe the types of treatments that have been attempted thus far, and delineate possible a stroke-like protocol that can be implemented in order to restore perfusion and recover vision after such ischemic complications have occurred.

## Introduction

The use of injectable cosmetic fillers has rapidly become increasingly popular in recent years—dermal fillers now represent the second most common nonsurgical aesthetic procedure (1). In 2021 alone, consumer spending on injectable cosmetic fillers in the United States amounted to over $1 billion (2). With this increase in injections comes an increase in retrograde embolism of cosmetic fillers and ophthalmic ischemia (3, 4).

Filler emboli have been shown to induce ophthalmic artery occlusion (OAO), central retinal artery occlusion (CRAO), branch retinal artery occlusion (BRAO), and other orbital vascular occlusions (5–7). Previous studies have established that permanent retinal damage resulting in infarction and vision loss occurs with as little as 12-15 minutes of non-perfusion, suggesting that timely intervention is key when vascular occlusion occurs. Additionally, studies show that many CRAO’s are incomplete and may still benefit from therapy after longer intervals (6, 7). Presently, there is no first-line protocol for rapid reperfusion in ischemic filler complications to reverse the vision changes associated with ocular ischemia. This article will review current proposed reperfusion techniques and discuss possible stroke-like protocols for addressing the ischemic complications of dermal filler injection.

## Mechanism of ophthalmic complications of filler injection

**Figure 1:**
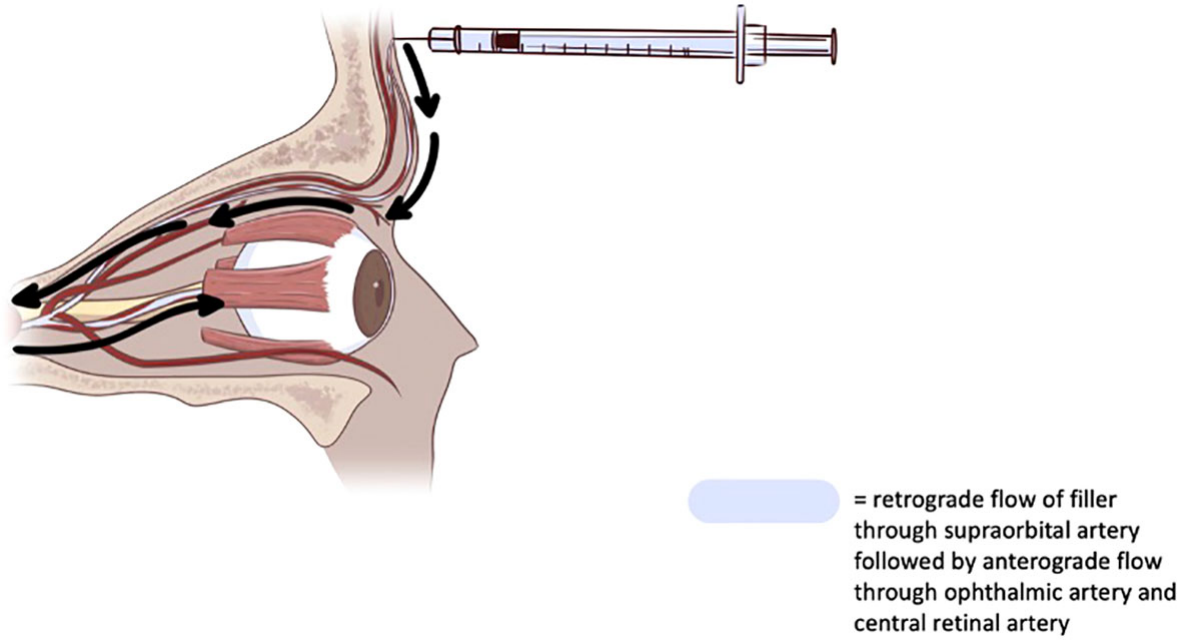
Diagram depicting retrograde embolism of cosmetic filler to ophthalmic artery.

The main proposed pathophysiology of ophthalmic injury following injection of cosmetic filler is retrograde embolism through an artery adjacent to the injection site, most commonly the supratrochlear, supraorbital, or dorsal nasal arteries, which are derived from the internal carotid artery (**Figure 1**). These arteries also anastomose with the external carotid artery via the ophthalmic artery. The supratrochlear and supraorbital arteries can inadvertently be infiltrated when injecting filler into the glabellar region, while the dorsal nasal artery is more likely to be accessed over the bridge of the nose (8). When the filler material is injected into one of these distal arteries, the relative higher pressure of the injection compared to arterial pressure causes the filler to travel proximally towards the internal carotid, where it can embolize to the ophthalmic artery (9, 10). Ischemia occurs due to primary obstruction of blood flow by the embolus itself and secondary triggering of local inflammation, platelet aggregation, and activation of the coagulation cascade. Lastly, larger emboli and clots can break into smaller micro-emboli and lodge in distal branches of the ophthalmic artery, causing multifocal vessel occlusion.

The ischemic consequences of filler-induced embolism can differ depending on the anatomic location affected. If the filler embolus lodges in the central retinal and posterior ciliary arteries, occlusion causes non-perfusion of the retina manifesting as vision loss that can become permanent once irreversible retinal cell death occurs (10).Occlusion of the posterior ciliary arteries and anterior ciliary arteries can limit blood flow to the iris and ciliary body, causing anterior segment ischemia, as well (11).

The type of cosmetic filler that is administered can also affect the extent of embolism and the ischemic sequelae that occur. Previously, fillers implicated in reports of visual complications have included hyaluronic acid, platelet-rich plasma (PRP), autologous fat, calcium hydroxyapatite, and periorbital aesthetic poly-(L)-lactic acid (PLLA) (12, 13). Autologous fat has been demonstrated to be more likely to induce a proximal arterial obstruction given its relatively larger particle size. Hyaluronic acid has been thought to occlude more distal branches of the ophthalmic artery due to its smaller particle size and can possibly also block perfusion by drawing water into adjacent soft tissues and thereby reducing the pressure gradient between the ophthalmic artery and its smaller branches. Platelet-rich plasma (PRP) can cause more severe ischemia since it is more pro-thrombotic than other fillers due to its composition—a highly concentrated amalgamation of a patient’s own platelets and associated growth factors, 2.5-8 times higher than normal serum concentration (14, 15).

## Treatment of ophthalmic filler embolism

In any case of embolic vascular occlusion, treatment requires reestablishing perfusion and oxygenation of the affected tissue. This can be achieved by adjuvant therapies that focus on clot and embolus displacement or increased oxygen tension, thrombolytic therapies that break down fibrin clot, or specific embolism lysis agents. None of the therapies described below have demonstrated efficacy from randomized data - this is expected given the current rarity of filler embolism. Even so, they warrant discussion because ischemia requires rapid reperfusion to restore function based on retinal models and any future trials will depend on rapid treatment protocols similar to those developed in the stroke and cardiovascular literature.

## Local administration of reversal agents

Hyaluronic acid is the only cosmetic filler with a specific treatment, hyaluronidase, which facilitates enzymatic degradation of the filler. Previous studies have examined the efficacy of injecting hyaluronidase via subcutaneous, retrobulbar, or intra-arterial approaches in an effort to reverse ischemia (16). Subcutaneously administered hyaluronidase can pass through tissue and diffuse across the arterial wall. A systematic review of 144 cases of using hyaluronidase to manage vision loss associated with hyaluronic-acid injection revealed varying success in improving in visual acuity, even when injection occurred immediately after vision loss (17). However, multiple patients demonstrated visual recovery when hyaluronidase was injected adjacent to the supraorbital or supratrochlear arteries, which have been implicated as possible areas of retrograde embolism of filler, further supporting this theory. Intra-arterial injection of hyaluronidase also resulted in partial visual recovery in multiple patients, suggesting that future research could examine the efficacy of rapid administration either directly into or next to the likely site of retrograde embolism to prevent permanent vision damage. With retrobulbar injection of hyaluronidase, only one patient experienced improvement in visual acuity and it was questionable whether her clinical recovery could be attributed to hyaluronidase injection or resolution of the corneal edema she exhibited at initial presentation (17). Though there are limited reports of successfully using hyaluronidase to break down displaced hyaluronic acid filler, further research could examine the efficacy of administering hyaluronidase immediately after initial vision loss as well as the utility of devising reversal agents for other types of cosmetic fillers.

## Potential use of thrombolytics or antiplatelet medications

Urokinase, tissue plasminogen activator (tPA), and tenecteplase (TNK) are thrombolytic agents that activate plasmin, which breaks down fibrin and fibrinogen, the final common components of the coagulation cascade. Intravenous administration of tPA or TNK is the current mainstay of endogenous stroke management and could be employed to treat iatrogenic ophthalmic artery occlusion following cosmetic filler embolism. Previous studies have revealed that filler-induced clots may induce a higher degree of vasospasm and thrombosis than those produced by endogenous emboli due to a combination of occlusion by filler embolus and platelets and fibrin that are drawn to the area of occlusion as part of the inflammatory and thrombotic response. Thus, clot dissolution should also target the endogenous embolus coupled with the filler embolus, instead of solely addressing dissolution of the embolized filler (18, 19). There are not yet any reported cases of injecting intravenous tPA after retrograde embolism of cosmetic filler, but urokinase has been used in filler embolism and there are various meta-analyses of the utility of tPA in endogenous CRAO (10, 20). In a meta-analysis of intravenous fibrinolysis for CRAO, Mac Grory et al. examined 67 patients who received tPA less than 4.5 hours after CRAO and noted visual recovery in 37.3%, which is higher than the rate of recovery of 17.7% previously noted in patients who did not undergo any therapeutic intervention (21).

Studies investigating the utility of intra-arterial thrombolysis to treat non-iatrogenic CRAO suggest variable efficacy (22) A systematic meta-analysis published by Page et al. revealed an odds ratio of 2.52 with a 95% confidence interval (1.69 – 3.77) and P < 0.0001 that intra-arterial tPA can increase visual recovery after CRAO (23). Similarly, a meta-analysis by Beatty et al. also indicated that 34.9% of patients who received intra-arterial therapy demonstrated significant improvement in visual acuity (24). Many such studies are limited in interpretation given the large variability in time to treatment and the fact that many endogenous CRAO’s can self-resolve with time. Also, most of these studies focused on visual acuity as a marker of clinical improvement though additionally assessing visual fields could provide more information about recovery of perfusion to areas of the retina beyond the fovea.

In terms of intra-arterial thrombolysis in the setting of filler-induced vision loss, a paper by Zhang et. alexamined the utility of IAT with urokinase in combination with hyaluronidase specifically in patients who experienced vision loss after hyaluronic acid filler embolism (10). In this paper, 10 out of 24 patients treated with either hyaluronidase or combined hyaluronidase and urokinase experienced improvement in visual acuity. While 36% of the patients who received intra-arterial hyaluronidase exclusively exhibited visual recovery, 46% of patients who received both hyaluronidase and urokinase experienced improvement in visual acuity. In another study, combining hyaluronidase with urokinase resulted in 100% of patients having either partial or complete reperfusion of the occluded artery with varying degrees of visual recovery (25, 26). These findings suggest that intra-arterial thrombolysis with a combination of agents, such as urokinase and hyaluronidase together, rather than one reversal agent alone could maximize the restoration of vision after filler embolism, though validation via randomized controlled trial is necessary to vet IAT as a viable treatment option.

**Table 1** includes a compilation of all reported cases of attempted intra-arterial thrombolysis (IAT) to treat cosmetic filler-related embolism and vision loss. To date, IAT has only been attempted in patients who received fillers composed of hyaluronic acid or fat. For the most part, improvement in visual acuity was generally minimal, though time to treatment was usually several hours after initial loss of vision, which is well past the time limit for irreversible retinal ischemia and could explain the variable success in instances of IAT after filler-related embolism. One study actually showed minimal to no restoration in vision even if there was evidence of successful reperfusion for the central retinal artery (35).

**Table 1:** Recent reports of intra-arterial thrombolysis in the setting of filler embolism.

Author	Age (years)	Sex	Material	Pre-Treatment Visual Acuity	Time to Treatment After Symptom Onset (hours)	Thrombolytic Agent	Visual Acuity Outcome
Kim et. al, 2016 (27)	19	Female	Hyaluronic acid	NLP, unilateral	N/A	Hyaluronidase	NLP, unilateral
Chen et. al, 2018 (28)	20	Female	Hyaluronic acid	Vision loss	2	Hyaluronidase, urokinase	NLP, OD
Oh et. al, 2014 (29)	33	Female	Hyaluronic acid	NLP, OD	10	Hyaluronidase, urokinase	NLP, OD
Kim et. al, 2015 (30)	24	Female	Hyaluronic acid	NLP, OD	3.5	Hyaluronidase	NLP, OD
Kim et. al, 2015 (30)	34	Female	Hyaluronic acid	NLP, OD	5	Hyaluronidase	NLP, OD
Kim et. al, 2015 (30)	39	Female	Hyaluronic acid	HM, OS	3	Hyaluronidase	NLP, OD
Kim et. al, 2015 (30)	41	Female	Hyaluronic acid	NLP, OD	4.5	Hyaluronidase	NLP, OD
Kim et. al, 2015 (30)	40	Female	Fat	NLP, OS	2.5	Urokinase, tirofiban	NLP, OS
Kim et. al, 2015 (30)	66	Female	Fat	NLP, OS	3	Urokinase	NLP, OS
Kim et. al, 2015 (30)	40	Female	Fat	NLP, OD	5	Urokinase	NLP, OD
Zhang et. al, 2020 (10)	N/A	Female	Hyaluronic acid	LP, OD	168	Hyaluronidase, urokinase	HM, OD
Zhang et. al, 2020 (10)	N/A	Female	Hyaluronic acid	NLP, OS	2	Hyaluronidase, urokinase	LP, OS
Zhang et. al, 2020 (10)	N/A	Female	Hyaluronic acid	NLP, OS	27	Hyaluronidase, urokinase	NLP, OS
Zhang et. al, 2020 (10)	N/A	Female	Hyaluronic acid	NLP, OS	17	Hyaluronidase, urokinase	NLP, OS
Zhang et. al, 2020 (10)	N/A	Female	Hyaluronic acid	LP, OD	6	Hyaluronidase, urokinase	20/200, OD
Zhang et. al, 2020 (10)	N/A	Female	Hyaluronic acid	NLP, OD	25	Hyaluronidase, urokinase	20/50, OD
Zhang et. al, 2020 (10)	N/A	Female	Hyaluronic acid	NLP, OS	16	Hyaluronidase, urokinase	20/133, OS
Zhang et. al, 2020 (10)	N/A	Female	Hyaluronic acid	LP, OS	26	Hyaluronidase, urokinase	20/50, OS
Zhang et. al, 2020 (10)	N/A	Female	Hyaluronic acid	NLP, OS	51	Hyaluronidase, urokinase	NLP, OS
Zhang et. al, 2020 (10)	N/A	Female	Hyaluronic acid	NLP, OD	22	Hyaluronidase, urokinase	NLP, OD
Zhang et. al, 2020 (10)	N/A	Female	Hyaluronic acid	NLP, OS	19	Hyaluronidase, urokinase	NLP, OS
Zhang et. al, 2020 (10)	N/A	Female	Hyaluronic acid	NLP, OS	46	Hyaluronidase, urokinase	NLP, OS
Zhang et. al, 2020 (10)	N/A	Male	Hyaluronic acid	NLP, OS	75	Hyaluronidase, urokinase	NLP, OS
Zhang et. al, 2020 (10)	N/A	Female	Hyaluronic acid	NLP, OS	20.5	Hyaluronidase	NLP, OS
Zhang et. al, 2020 (10)	N/A	Female	Hyaluronic acid	LP, OS	24	Hyaluronidase	HM, OS
Zhang et. al, 2020 (10)	N/A	Female	Hyaluronic acid	LP, OS	144	Hyaluronidase	20/40, OS
Zhang et. al, 2020 (10)	N/A	Female	Hyaluronic acid	NLP, OD	24	Hyaluronidase	20/50, OD
Zhang et. al, 2020 (10)	N/A	Female	Hyaluronic acid	NLP, OD	24	Hyaluronidase	NLP, OD
Zhang et. al, 2020 (10)	N/A	Female	Hyaluronic acid	NLP, OD	32	Hyaluronidase	NLP, OD
Zhang et. al, 2020 (10)	N/A	Female	Hyaluronic acid	NLP, OD	36	Hyaluronidase	NLP, OD
Zhang et. al, 2020 (10)	N/A	Female	Hyaluronic acid	NLP, OS	100	Hyaluronidase	NLP, OS
Zhang et. al, 2020 (10)	N/A	Female	Hyaluronic acid	NLP, OD	14	Hyaluronidase	LP, OD
Zhang et. al, 2020 (10)	N/A	Female	Hyaluronic acid	NLP, OD	24	Hyaluronidase	NLP, OD
Zhang et. al, 2020 (10)	N/A	Female	Hyaluronic acid	NLP, OS	48	Hyaluronidase	NLP, OS
Zhang et. al, 2021 (31)	61	Female	Hyaluronic acid	Blurred vision, OD	26	Hyaluronidase	Blurred vision, OD
Zhang et. al, 2021 (31)	31	Female	Hyaluronic acid	NLP, OD	4	Hyaluronidase	LP, OD
Zhang et. al, 2021 (31)	31	Female	Hyaluronic acid	Blurred vision, OS	6	Hyaluronidase	Blurred vision, OS
Zhang et. al, 2021 (31)	46	Female	Hyaluronic acid	NLP, OD	5	Hyaluronidase	NLP, OD
Nguyen et. al, 2022 (32)	27	Female	Hyaluronic acid	NLP, OD	4	Hyaluronidase	20/50, OD
Wang et. al, 2021 (33)	18 -35	29 females, 1 male	Hyaluronic acid	27 individuals with NLP vision in affected eye, 3 individuals with bare LP in affected eye	20 - 120	Hyaluronidase	Visual acuity improvement in 9 individuals
Xu et al, 2021 (34)	24	Female	Hyaluronic acid	NLP, OS	6	Hyaluronidase, papaverine	NLP, OS
Xu et al, 2021 (34)	24	Female	Hyaluronic acid	NLP, OS	7	Hyaluronidase, papaverine	NLP, OS
Xu et al, 2021 (34)	35	Female	Hyaluronic acid	NLP, OS	10	Hyaluronidase, papaverine	NLP, OS
Xu et al, 2021 (34)	29	Female	Hyaluronic acid	NLP, OS	8	Hyaluronidase, papaverine	NLP, OS
Xu et al, 2021 (34)	19	Female	Hyaluronic acid	LP, OD	2	Hyaluronidase, papaverine	HM, OD
Xu et al, 2021 (34)	21	Female	Hyaluronic acid	NLP, OS	5	Hyaluronidase, papaverine	NLP, OS
Xu et al, 2021 (34)	19	Male	Hyaluronic acid	NLP, OS	6	Hyaluronidase, papaverine	LP, OS
Xu et al, 2021 (34)	35	Female	Hyaluronic acid	NLP, OD	5	Hyaluronidase, papaverine	LP, OD
Zhang et al, 2023 (35)	N/A	Female	Hyaluronic acid	Blurred vision, OD	26	Hyaluronidase	Blurred vision, OD
Zhang et al, 2023 (35)	N/A	Female	Hyaluronic acid	NLP, OD	4	Hyaluronidase	NLP, OD
Zhang et al, 2023 (35)	N/A	Female	Hyaluronic acid	Blurred vision, OS	6	Hyaluronidase	Blurred vision, OS
Zhang et al, 2023 (35)	N/A	Female	Hyaluronic acid	NLP, OS	5	Hyaluronidase	NLP, OS

One conceivable concern regarding thrombolysis with tPA is risk of inducing a hemorrhage or other possible, related systemic adverse effects. With regards to intravenous tPA use, previous research has demonstrated that the risk of inducing a hemorrhage by administering intravenous tPA to patients without a confirmed stroke is low. In a large multi-center study of 75,582 patients, the rate of symptomatic intracranial hemorrhage was 3.5% in stroke patients versus 0.4% in stroke mimics (36). These findings suggest that given minimal risk of disastrous hemorrhagic effects, clinicians should consider the potential utility of performing intravenous thrombolysis with tPA in filler embolism. Additional research could investigate the safety of intra-arterial tPA via the site of filler injection as a therapeutic option, as this would allow for localized administration of thrombolytic and the complications that have previously limited intra-arterial thrombolysis in stroke are related to intracerebral hemorrhage and vascular injury from endovascular therapy (22, 37). For example, one paper investigated treating CRAO with intra-retinal arterial cannulation using a microneedle to precisely administer tPA to the central retinal artery via vitrectomy, and outcomes were evaluated using fluorescein angiography, demonstrating complete restoration of blood flow in 10 eyes and partial restoration of blood flow in 3 eyes (38). Another paper described intra-arterial cannulation of the supratrochlear and supraorbital arteries and injection of urokinase and papaverine to manage CRAO in 26 patients via cut-down and blunt dissection with 92.3% efficacy rate (39). A case series to highlight included 17 patients who underwent intra-arterial hyaluronidase injected into the facial artery or supratrochlear arteries via a percutaneous approach with color doppler imaging, resulting in reversal of skin necrosis in all patients (40). This method is more accessible in an outpatient or office setting.

Other studies also suggest that embolism associated with PRP may be more resistant to recanalization via tPA compared to endogenous emboli because tPA targets and promotes lysis of fibrin rather than directly targeting the platelets themselves (23). While antiplatelet agents are a mainstay of endogenous stroke therapy, their benefit is largely limited to the prevention of recurrent stroke (41).

## Systemic management and adjuvant therapies

Besides interventions localized to the injection site, multiple systemic and adjuvant therapies have been attempted both in the setting of endogenous and iatrogenic filler-associated central retinal artery occlusion. Many of these therapeutic methods focus on lowering intraocular pressure (IOP) to manipulate the pressure gradient within the eye and shift emboli to more distal vessels and preserve central vision. IOP-lowering treatments including anterior chamber paracentesis, topical anti-glaucoma drops (including beta-adrenergic blocker, carbonic anhydrase inhibitor, prostaglandin analogue, and alpha agonist) and intravenous acetazolamide have been proposed (42). Intravenous mannitol can also be used to create a larger gradient between ischemic tissue pressure and arterial pressure. The use of intravenous glyceryl trinitrate has similarly been thought to help alter the pressure gradient by lowering retinal venous pressure, though studies have also raised concern for potential exacerbation of ischemia secondary to reduced perfusion or side effects such as dizziness or fainting (43–45).Lastly, ocular massage has been reported to lower intraocular pressure and potentially dislodge emboli (42, 46).

To target the initial inflammatory response and edematous sequelae of retinal ischemia, high-dose systemic glucocorticoids, such as IV methylprednisolone, can be administered to help manage the consequences of CRAO induced by filler embolism (10). Finally, in some cases, hyperbaric oxygen therapy has been utilized to enhance oxygen delivery to areas of ischemia within the retina until hypoperfusion resolves (42). However, none of these methods have been validated as a first-line treatment for either endogenous or iatrogenic CRAO, so future randomized control trials could also help determine what kind of role these adjuvant therapies could play in treating cosmetic filler embolism.

## Stroke-like protocols

Reversal agents for specific filler materials, re-vascularization using thrombolysis, and systemic and ocular therapies to promote ocular reperfusion all require early intervention. While the efficacy of these therapies has yet to be shown in randomized trials, the need for rapid assessment, early intervention, and post-treatment monitoring suggest that visual complications of cosmetic filler injection should be approached with a stroke-like protocol.

One of the main determinants of resolution of visual impairment is timely intervention—in animal models, rectifying at least some retinal damage has been shown to be possible if blood flow was restored in less than 90 minutes, though irreversible vision loss can be seen within 12-15 minutes after embolism. After 100 minutes, there is variability in the amount of retinal recovery and once 4 hours have passed, non-perfusion of the retina results in profound, irreversible infarction (47). As soon as vision loss and any other focal neurological deficits are noted after injection of cosmetic filler, patients should be referred emergently to a stroke center capable of further neurologic and ophthalmic evaluation and possible revascularization if deemed necessary. Similar to a regular stroke work-up, patients should urgently undergo head computed tomography without contrast to rule out intracranial hemorrhage and head computed tomography with angiography to assess for specific vascular occlusions amenable to thrombectomy. If no alternative diagnoses are identified, revascularization can be attempted with systemic therapies and reversal agents specific to the type of filler used. If no contraindications to thrombolysis are identified, intravenous tPA could be administered to promote fibrinolysis.

An evidence and consensus-based protocol is being developed to help injectors manage vision-threatening complications of filler embolism and the preliminary guidelines are delineated below.

1. Preparation –

- Have a direct contact to a local stroke team and any sub-specialists who can be readily available, with signs posted and staff trained.
- Share a protocol/paper for intra-arterial filler dissolution with the stroke team.^32^
- Keep a minimum of 1500U of hyaluronidase in the office at all times with a plan of how to urgently access more as needed.
- Obtain a baseline visual acuity and photos.
- Recognize ischemia by pain or pallor (no epinephrine in any anesthetic block if used)
- Immediately stop injecting and plan to treat with hyaluronidase as described below, repeating injections every 15 minutes.
- Have a team member immediately call emergency services (e.g. 911) to activate stroke team and sub-specialists for urgent transfer to a stroke hospital. While patient is being prepared for transfer, initiate treatment with ocular massage and hyaluronidase as below and accompany the patient during the transfer to continue the treatment en route to the stroke hospital.
- Initiate ocular massage and instruct patient to continue massage with circular, continuous pressure over closed eye cycling between massaging for 5 seconds, then resting for 5 seconds
- Flood the supraorbital/supratrochlear region with hyaluronidase with 600-1600U (attempting intra-arterial administration if possible through the supraorbital/supratrochlear foramina/notches). Percutaneous intra-arterial hyaluronidase through the facial, supraorbital, supratrochlear or angular arteries can also be attempted. Peribulbar or retrobulbar administration of hyaluronidase can be considered by an ophthalmologist as well if no additional risk; there is no clear evidence that the hyaluronidase can cross the blood-brain barrier into the central retinal artery.
- Consider adjuvant treatments including oral aspirin, intraocular pressure lowering agents or anterior chamber paracentesis, IV or oral steroids, hyperbaric oxygen, and vasodilators such phosphodiesterase inhibitors (e.g. sildenafil).

A more detailed protocol is in development to equip injectors and stroke teams to manage vision-threatening complications of fillers.

## Conclusions

Over the years, the administration of cosmetic fillers with compositions including hyaluronic acid, fat, and platelet-rich plasma has risen significantly in popularity. Concurrently, there has been an increase in the number of cases of visual complications following filler administration. Protocols for assessing and treating ophthalmic filler emboli must take into account the need for emergent reperfusion in order to salvage tissue. Thrombolytic therapy has been successfully tried in some case series via endovascular approaches with the most success in those who presented early. Though there are no proven therapies for cosmetic filler emboli, we believe that treatment should include immediate injection of reversal agents (such as injection of hyaluronidase, ideally intra-arterial) and use of adjuvant therapies, immediate referral to a stroke center for possible thrombolytic therapy, neurologic and ophthalmologic assessment, non-contrasted and contrasted head imaging. Planning ahead for these potential complications, being equipped with hyaluronidase and adjuvant therapies, and being connected to local resources and collaborators to rapidly refer these patients is critical. Protocolized care will not only improve treatment times, but also support further research on the efficacy of specific therapies.

## References

[1] Akinbiyi T Othman S Familusi O Calvert C Card EB Percec I. Better results in facial rejuvenation with fillers. Plast Reconstr Surg Glob Open. (2020) 8:e2763. doi: 10.1097/GOX.0000000000002763 33173655 PMC7647625

[2] Aesthetic Society. Available online at: https://cdn.theaestheticsociety.org/media/statistics/2021-TheAestheticSocietyStatistics.pdf (Accessed September 10, 2023).

[3] Rauso R Sesenna E Fragola R Zerbinati N Nicoletti GF Tartaro G. Skin necrosis and vision loss or impairment after facial filler injection. J Craniofac Surg. (2020) 31:2289–93. doi: 10.1097/SCS.0000000000007047 33136873

[4] Mehta P Kaplan JB Zhang-Nunes S. Ischemic complications of dermal fillers. Plast Aesthetic Res. (2022) 9:57. doi: 10.20517/2347-9264.2022.19

[5] Rohrich RJ Bartlett EL Dayan E. Practical approach and safety of hyaluronic acid fillers. Plast Reconstr Surg Glob Open. (2019) 7:e2172. doi: 10.1097/GOX.0000000000002172 31624663 PMC6635180

[6] Hayreh SS Kolder HE Weingeist TA. Central retinal artery occlusion and retinal tolerance time. Ophthalmology. (1980) 87:75–8. doi: 10.1016/s0161-6420(80)35283-4 6769079

[7] Tobalem S Schutz JS Chronopoulos A. Central retinal artery occlusion - rethinking retinal survival time. BMC Ophthalmol. (2018) 18:101. doi: 10.1186/s12886-018-0768-4 29669523 PMC5907384

[8] Kim EG Eom TK Kang SJ. Severe visual loss and cerebral infarction after injection of hyaluronic acid gel. J Craniofac Surg. (2014) 25:684–6. doi: 10.1097/SCS.0000000000000537 24621723

[9] Yi KH Lee HJ Kim WR An MH Park HJ Hu H. Does injecting small amounts of fillers prevent the development of secondary blindness? J Cosmet Dermatol. (2024) 1:84–9. doi: 10.1111/jocd.15898 37381604

[10] Zhang LX Lai LY Zhou GW Liang LM Zhou YC Bai XY. Evaluation of intraarterial thrombolysis in treatment of cosmetic facial filler-related ophthalmic artery occlusion. Plast Reconstr Surg. (2020) 145:42e–50e. doi: 10.1097/PRS.0000000000006313 31881603

[11] Borruat FX Bogousslavsky J Uffer S Klainguti G Schatz NJ. Orbital infarction syndrome. Ophthalmology. (1993) 100:562–8. doi: 10.1016/s0161-6420(93)31606-4 8479716

[12] Oh DJ Jiang Y Mieler WF. Ophthalmic artery occlusion and subsequent retinal fibrosis from a calcium hydroxylapatite filler injection. J Vitreoretin Dis. (2019) 3:190–3. doi: 10.1177/2474126419834782 PMC6533913 31131349

[13] Roberts SAI Arthurs BP. Severe visual loss and orbital infarction following periorbital aesthetic poly-(L)-lactic acid (PLLA) injection. Ophthal Plast Reconstr Surg. (2012) 28:e68–70. doi: 10.1097/IOP.0b013e3182288e4d 21862947

[14] Seth R Kang RS Lee MK Keller GS. Platelet-rich plasma in cosmetic surgery. Int J Otorhinolaryngol Clin. (2013) 5:24–8. doi: 10.5005/jp-journals-10003-1106

[15] Tomkins AJ Schleicher N Murtha L Kaps M Levi CR Nedelmann M. Platelet rich clots are resistant to lysis by thrombolytic therapy in a rat model of embolic stroke. Exp Transl Stroke Med. (2015) 7:2. doi: 10.1186/s13231-014-0014-y 25657829 PMC4318170

[16] Kim DW Yoon ES Ji YH Park SH Lee BI Dhong ES. Vascular complications of hyaluronic acid fillers and the role of hyaluronidase in management. J Plast Reconstr Aesthetic Surg JPRAS. (2011) 64:1590–5. doi: 10.1016/j.bjps.2011.07.013 21807574

[17] Xiao H Kou W Yang Y Dai EK Zhang XR Wen YJ. Administration method and potential efficacy of hyaluronidase for hyaluronic acid filler-related vision loss: A systematic review. Aesthetic Plast Surg. (2024) 4:709–18. doi: 10.1007/s00266-022-03215-9 36574028

[18] Baley-Spindel I Villaseñor-Villalpando E Márquez-Espriella C Rivera-Salgado MI Dávila-Díaz R. Perivascular hyaluronidase with alteplase as treatment for hyaluronic acid thrombosis. Aesthet Surg J. (2020) 40:551–9. doi: 10.1093/asj/sjz101 30957144

[19] Stark K Massberg S. Interplay between inflammation and thrombosis in cardiovascular pathology. Nat Rev Cardiol. (2021) 18:666–82. doi: 10.1038/s41569-021-00552-1 PMC8100938 33958774

[20] Yang H Zheng Y Li D Li TZ Zhao JH Shu KY. Facial artery branch thrombolysis for nasal vascular embolism induced by hyaluronic acid injection. Ann Plast Surg. (2024) 93:658–63. doi: 10.1097/SAP.0000000000004136 39526813

[21] Mac Grory B Nackenoff A Poli S Spitzer MS Nedelmann M Guillon B. Intravenous fibrinolysis for central retinal artery occlusion: A cohort study and updated patient-level meta-analysis. Stroke. (2020) 51:2018–25. doi: 10.1161/STROKEAHA.119.028743 32568646

[22] Hakim N Hakim J. Intra-arterial thrombolysis for central retinal artery occlusion. Clin Ophthalmol Auckl NZ. (2019) 13:2489–509. doi: 10.2147/OPTH.S232560 PMC6916701 31853171

[23] Page PS Khattar NK White AC Cambon AC Brock GN Rai SN. Intra-arterial thrombolysis for acute central retinal artery occlusion: A systematic review and meta-analysis. Front Neurol. (2018) 9:76. doi: 10.3389/fneur.2018.00076 29527185 PMC5829526

[24] Beatty S Eong K. Local intra-arterial fibrinolysis for acute occlusion of the central retinal artery: a meta-analysis of the published data. Br J Ophthalmol. (2000) 84:914–6. doi: 10.1136/bjo.84.8.914 PMC1723600 10906103

[25] Wang Y Li Q Ye Y. Intraarterial thrombolytic treatment for visual deficits caused by hyaluronic acid filler: efficacy, safety, and prognostic factors. Plast Reconstr Surg. (2023) 152:1226. doi: 10.1097/PRS.0000000000010374 36877754 PMC10666935

[26] Schrag M Youn T Schindler J Kirshner H Greer D. Intravenous fibrinolytic therapy in central retinal artery occlusion: A patient-level meta-analysis. JAMA Neurol. (2015) 72:1148–54. doi: 10.1001/jamaneurol.2015.1578 26258861

[27] Kim A Kim SH Kim HJ Yang HK Hwang JM Kim JS. Ophthalmoplegia as a complication of cosmetic facial filler injection. Acta Ophthalmol (Copenh). (2016) 94:e377–9. doi: 10.1111/aos.12893 26407823

[28] Chen YC Wu HM Chen SJ Lee HJ Lirng JF Lin CJ. Intra-arterial thrombolytic therapy is not a therapeutic option for filler-related central retinal artery occlusion. Facial Plast Surg FPS. (2018) 34:325–9. doi: 10.1055/s-0037-1621730 29378380

[29] Oh BL Jung C Park KH Hong YJ Woo SJ. Therapeutic intra-arterial hyaluronidase infusion for ophthalmic artery occlusion following cosmetic facial filler (Hyaluronic acid) injection. Neuro-Ophthalmol Aeolus Press. (2014) 38:39–43. doi: 10.3109/01658107.2013.830134 PMC5123061 27928273

[30] Kim YK Jung C Woo SJ Park KH. Cerebral angiographic findings of cosmetic facial filler-related ophthalmic and retinal artery occlusion. J Korean Med Sci. (2015) 30:1847–55. doi: 10.3346/jkms.2015.30.12.1847 PMC4689831 26713062

[31] Zhang L Luo Z Li J Liu Z Xu H Wu MQ. Endovascular hyaluronidase application through superselective angiography to rescue blindness caused by hyaluronic acid injection. Aesthet Surg J. (2021) 41:344–55. doi: 10.1093/asj/sjaa036 32401308

[32] Nguyen HH Tran HTT Duong QH Nguyen MD Dao HX Le DT. Significant vision recovery from filler-induced complete blindness with combined intra-arterial injection of hyaluronidase and thrombolytic agents. Aesthetic Plast Surg. (2022) 46:907–11. doi: 10.1007/s00266-021-02658-w 34767060

[33] Wang J Shen H Liu T Li Q Lyu Z Yu Y. An efficacy and safety study of intra-arterial recanalization of occluded ophthalmic arteries in patients with monocular blindness caused by injection of hyaluronic acid in facial tissues. Aesthetic Plast Surg. (2021) 45:1573–8. doi: 10.1007/s00266-021-02224-4 33770216

[34] Xu X Zhou G Fu Q Zhang LX Yu YT Dong Y. Efficacy of intra-arterial thrombolytic therapy for vision loss resulting from hyaluronic acid filler embolization. J Cosmet Dermatol. (2021) 20:3205–12. doi: 10.1111/jocd.14111 PMC8596545 33825341

[35] Zhang L Zhou Q Xu H Gu QH Shi HY Pan L. Long-term prognosis of vision loss caused by facial hyaluronic acid injections and the potential approaches to address this catastrophic event. Aesthet Surg J. (2023) 43:484–93. doi: 10.1093/asj/sjac329 36495213

[36] Ali-Ahmed F Federspiel JJ Liang L Xu H Sevilis T Hernandez AF. Intravenous tissue plasminogen activator in stroke mimics. Circ Cardiovasc Qual Outcomes. (2019) 12:e005609. doi: 10.1161/CIRCOUTCOMES.119.005609 31412730 PMC6699639

[37] Lee M Hong KS Saver JL. Efficacy of intra-arterial fibrinolysis for acute ischemic stroke. Stroke. (2010) 41:932–7. doi: 10.1161/STROKEAHA.109.574335 20360549

[38] Kadonosono K Yamane S Inoue M Yamakawa T Uchio E. Intra-retinal arterial cannulation using a microneedle for central retinal artery occlusion. Sci Rep. (2018) 8:1360. doi: 10.1038/s41598-018-19747-7 29358594 PMC5778058

[39] Wang R Qian L Wang Y Zheng Y Du SS Lei T. Evaluation of ophthalmic artery branch retrograde intervention in the treatment of central retinal artery occlusion (CRAO). Med Sci Monit Int Med J Exp Clin Res. (2017) 23:114. doi: 10.12659/MSM.898352 PMC5240882 28064304

[40] Zheng C Fu Q Zhou Gw Lai LY Zhang LX Zhang DQ. Efficacy of percutaneous intraarterial facial/supratrochlear arterial hyaluronidase injection for treatment of vascular embolism resulting from hyaluronic acid filler cosmetic injection. Aesthet Surg J. (2022) 42:649–55. doi: 10.1093/asj/sjab425 34958671

[41] Minhas JS Chithiramohan T Wang X Barnes SC Clough RH Kadicheeni M. Oral antiplatelet therapy for acute ischaemic stroke. Cochrane Database Syst Rev. (2022) 1:CD000029. doi: 10.1002/14651858.CD000029.pub4 35028933 PMC8758582

[42] Cugati S Varma DD Chen CS Lee AW. Treatment options for central retinal artery occlusion. Curr Treat Options Neurol. (2013) 15:63–77. doi: 10.1007/s11940-012-0202-9 23070637 PMC3553407

[43] Humzah MD Ataullah S Chiang C Malhotra R Goldberg R. The treatment of hyaluronic acid aesthetic interventional induced visual loss (AIIVL): A consensus on practical guidance. J Cosmet Dermatol. (2019) 18:71–6. doi: 10.1111/jocd.12672 PMC6912247 29885087

[44] Carley SK Kraus CN Cohen JL. Nitroglycerin, or not, when treating impending filler necrosis. Dermatol Surg Off Publ Am Soc Dermatol Surg Al. (2020) 46:31–40. doi: 10.1097/DSS.0000000000002030 31356437

[45] Hwang CJ Morgan PV Pimentel A Sayre JW Goldberg RA Duckwiler G. Rethinking the role of nitroglycerin ointment in ischemic vascular filler complications: an animal model with ICG imaging. Ophthal Plast Reconstr Surg. (2016) 32:118–22. doi: 10.1097/IOP.0000000000000446 25794030

[46] Svoronos AA Scott NL. Vision recovery after ocular massage for cosmetic filler-induced ophthalmic artery occlusion. Am J Ophthalmol Case Rep. (2024) 36:102229. doi: 10.1016/j.ajoc.2024.102229 39717359 PMC11665411

[47] Hayreh SS Weingeist TA. Experimental occlusion of the central artery of the retina. IV: Retinal tolerance time to acute ischaemia. British J Ophthalmol. (1980) 11:818–25. doi: 10.1136/bjo.64.11.818 PMC1043826 7426553

